# Mental Health and Recreational Angling in UK Adult Males: A Cross-Sectional Study

**DOI:** 10.3390/epidemiologia4030030

**Published:** 2023-07-13

**Authors:** Jason J. Wilson, Mike Trott, Mark A. Tully, Rosie K. Lindsay, Matt Fossey, Lauren Godier-McBard, Laurie T. Butler, Andy Torrance, Lee Smith

**Affiliations:** 1 Sport and Exercise Sciences Research Institute, School of Sport, Ulster University, Newtownabbey BT37 0QB, UK; 2 Centre for Public Health, School of Medicine, Dentistry and Biomedical Sciences, Queen’s University Belfast, Belfast BT12 6BA, UK; m.trott@qub.ac.uk; 3 Vision and Eye Research Institute, School of Medicine, Anglia Ruskin University, Cambridge CB1 1PT, UK; 4 School of Medicine, Ulster University, Londonderry BT48 7JL, UK; m.tully@ulster.ac.uk; 5 Vision and Hearing Sciences Research Centre, Anglia Ruskin University, Cambridge CB1 1PT, UK; rkl109@pgr.aru.ac.uk; 6 Veterans and Families Institute for Military Social Research, Anglia Ruskin University, Chelmsford CMI 1SQ, UK; matt.fossey@aru.ac.uk (M.F.); lauren.godier-mcbard@aru.ac.uk (L.G.-M.); 7 The Centre for Mental Health, London W1G 0AN, UK; 8 Faculty of Science and Engineering, Anglia Ruskin University, Cambridge CB1 1PT, UK; laurie.butler@aru.ac.uk; 9 Angling Direct PLC, Norfolk NR13 6LH, UK; 10 Centre for Health, Performance and Wellbeing, Anglia Ruskin University, Cambridge CB1 1PT, UK; lee.smith@aru.ac.uk

**Keywords:** fishing, angling, mental health, United Kingdom, survey

## Abstract

Exposure to aquatic environments (i.e., blue spaces) can lead to improved mental health and well-being. One meaningful way to spend time in blue spaces is through recreational angling, although limited scientific literature exists on this topic. The present study aims to examine the relationship between recreational angling and mental health and well-being in a sample of UK adult male anglers. A cross-sectional online survey asked questions about demographic characteristics, participation in recreational angling, physical activity levels, diagnosis of psychiatric disorders, and mental health and well-being. Relationships between angling status (i.e., how often and how long participants angled for) and mental health variables were determined using regression models adjusted for age. In total, 1752 participants completed the survey. The regression models found that those who took part in angling more regularly had reduced odds of having depression (*p* < 0.001), schizophrenia (*p* = 0.001), suicidal thoughts (*p* < 0.001), and deliberately self-harming (*p* = 0.012), in addition to having a higher mental well-being and lower symptoms of depression and anxiety compared to those taking part in angling less frequently. In general, the findings suggest that encouraging frequent participation in recreational angling could be a dual method strategy for promoting relaxation and positive mental health, as well as encouraging increased levels of physical activity in those with mental health issues.

## 1. Introduction

There is a considerable evidence base that demonstrates the impact of exposure to aquatic environments, known as blue spaces, for improved mental health and well-being [1]. These benefits may be realised by living near blue spaces or intentionally participating in activities within blue spaces [2]. The suggested pathways for these benefits include mitigation (e.g., less exposure to harmful environments), instauration (e.g., promoting beneficial health outcomes like improved self-confidence), and restoration (e.g., reduced stress) [2]. One way to spend meaningful time in a blue space is through recreational fishing/angling (forthwith referred to as recreational angling).

In 2019, an estimated 2.0% (approximately 1.25 million) of people had participated in some form of recreational angling in blues spaces across Great Britain [3], making angling one of the more popular leisure pursuits in the UK. With small adaptions, recreational angling is one of the few forms of outdoor recreation that can be enjoyed across the course of life [4]. Across industrialised countries, it was estimated that approximately 11% of the population participate in recreational angling [5]. Given this popularity, angling makes a significant contribution to society in terms of social cohesion and economics [6]. For example, it was estimated that freshwater fisheries in England provide GBP 1.7 billion annually to the economy through direct benefits such as spending on clothing and equipment, food, and travel [7]. There are also likely to be additional health benefits for individuals such as increased fitness, physical functioning, and the ability to do activities for daily living [6]. This has given rise to an increased interest in angling as a form of therapy, with some health trusts in England partnering with the “Tackling Minds” charity to start socially prescribing angling for individuals with anxiety and depression [8].

Despite the popularity of recreational angling and its potential utility to address the rising levels of mental health problems in the UK [9,10], there is limited peer-reviewed research on the status of mental health and well-being related to recreational angling. Previous research has found that angling can be a form of stress relief [11,12], can improve social relations [13], and appears to be an accessible form of physical activity for those with disabilities [14], older adults, and individuals recovering from illness [15]. In addition, angling could be a useful modality for engaging more males to be physically active as it was highlighted that they are under-represented in most physical activity intervention studies [16].

Therefore, the aim of this paper is to examine the relationship between recreational angling and mental health and well-being in a sample of UK adult male anglers. This has the potential to inform targeted mental health interventions, future research, and public health policy.

## 2. Materials and Methods

### 2.1. Study Design and Sample

A cross-sectional study design was utilised. Participants were recruited from October 2021 to January 2022 via an online survey (Figure S1) that was advertised through the Instagram, Facebook, and Twitter accounts of Angling Direct and Tackling Minds. Angling Direct also sent the survey link to their mailing list, and the link was distributed via the Anglia Ruskin University Twitter account, as well as the authors’ own networks. The survey was open to all UK residents aged 18 years and over. Participants provided informed consent before completing the survey, and ethical approval was granted by the Anglia Ruskin University Sport and Exercise Science Ethics Panel.

### 2.2. Measures

#### 2.2.1. Demographic Variables

Participants were asked to provide demographic variables including age, country they live in, ethnicity, relationship status, presence of disability, employment status, household income, smoking status, and alcohol consumption.

#### 2.2.2. Psychiatric Disorders

Participants were asked if they had any of the following diagnosed psychiatric disorders: anxiety disorder, depression, bipolar disorder, schizophrenia, or ‘any other psychiatric disorder’. Other questions included whether they have had any suicidal thoughts, have attempted suicide, or have deliberately self-harmed themselves.

#### 2.2.3. Mental Health and Well-Being

Overall well-being was assessed using the Warwick–Edinburgh Mental Well-being Scale (WEMWBS), which consists of 14 questions on a Likert scale. Total scores ranged from 14 to 70, with higher scores indicating greater levels of mental well-being. The WEMWBS is well validated across several populations [17,18,19]. Sub-clinical depression was examined using the Beck Depression Inventory (BDI), a 21-item questionnaire scored on a Likert scale. Total scores ranged from 0 to 63, with higher scores indicating higher levels of depression. The BDI is validated across several populations [20]. In terms of depressive symptoms, total BDI scores of 0–13 are considered minimal, 14–19 are mild, 20–28 are moderate, and 29–63 are severe [21]. Sub-clinical anxiety was examined using the Beck Anxiety Inventory (BAI), a 21-item questionnaire scored on a Likert scale. Total scores ranged from 0 to 63, with higher scores indicating higher anxiety levels. In terms of anxiety symptoms, total BAI scores of 0–7 are minimal, 8–15 are mild, 16–25 are moderate, and 26–63 are severe [22]. The BAI is validated across several clinical and non-clinical populations [23].

#### 2.2.4. Angling Status

Participants were asked if they participated in angling and, if so, asked how often they took part (i.e., from “every day” to “less than once every six months”) as well as how long they would participate in angling during a normal session (i.e., from “under one hour” to “five or more hours”).

#### 2.2.5. Sedentary Behaviour and Physical Activity

Participants were asked how long they sat each day as well as how much time they spent watching a screen. Participants were also asked how long they engaged in moderate (defined as activities that take moderate physical effort and make you breathe somewhat harder than normal) and vigorous (defined as activities that take hard physical effort and make you breathe much harder than normal) physical activity on an average day as well as how much time they typically spent outdoors.

### 2.3. Statistical Analysis

SPSS version 28.0 (IBM, Chicago, IL, USA) was used for conducting analyses. Descriptive categorical data are presented as numbers (percentage/%) and continuous data as medians (25th–75th interquartile range/IQR) unless stated otherwise. To determine if fishing status was associated with mental health and mental well-being outcomes, a logistic regression was conducted for dichotomous variables (anxiety disorder, depression, bipolar disorder, schizophrenia, and other psychiatric conditions), and a linear regression was conducted for continuous variables (WEMWBS, BDI, and BAI scores). Regression models were chosen as they are robust against non-parametric data. All regression models were adjusted for age. Statistical significance for regression analyses was set at *p* < 0.05.

## 3. Results

Originally, a total of 1792 participants completed the questionnaire; however, we only received a small number of female respondents (n = 40). Therefore, we only analysed the males in the sample (n = 1752). Table 1 highlights the characteristics of the cohort. The majority of the cohort was white, was married or in a cohabiting relationship, had no disability, was in employment, had a household income above GBP 25,000, did not smoke, and drank alcohol. The most prevalent mental health issue was suicidal thoughts (30.3%), followed by depression (23.2%) and anxiety disorder (16.1%). Less than 10% of the sample reported having one or more of the following: bipolar disorder (<1%); schizophrenia (<1%); other psychiatric disorders (<4%); suicide attempts (<7%); and having deliberately self-harmed (<10%).

Also in Table 1, in terms of fishing status, 70.3% of the sample angled at least once every two weeks, while over 98% of angling sessions lasted for at least three hours. For sedentary behaviour and physical activity, the median reported that daily sitting and screen times were each 2 h, while the median daily moderate and vigorous physical activity times were 1.5 h (IQR 1.0–4.0) and 1.0 h (IQR 1.0–2.0), respectively.

Regarding the logistic regression models displayed in Table 2, after adjusting for age, the psychiatric conditions which yielded a significant relationship (*p* < 0.05) with how often participants took part in angling included the presence of depression, schizophrenia, suicidal thoughts, and deliberate self-harm. These significant regressions stated that the odds of having depression were 1.172 (95% CI: 1.078 to 1.273), schizophrenia were 1.879 (95% CI: 1.177 to 2.997), suicidal thoughts were 1.162 (95% CI: 1.072 to 1.260), and deliberate self-harm were 1.166 (95% CI: 1.034 to 1.314), and they were less likely the more frequently participants took part in angling. In terms of the normal length of an angling session, the only significant relationship was with suicidal thoughts (odds ratio: 0.678 95% CI: 0.521 to 0.882), which conversely suggested that the odds of having suicidal thoughts were higher the longer the normal length of an angling session was.

Regarding the linear regression models displayed in Table 3, after adjusting for age, significant relationships highlighted (*p* < 0.05) with how often participants took part in angling included the WEMWBS, BDI, and BAI scores. These significant regressions showed that mental well-being was higher (β = −0.507; 95% CI −0.690 to −0.324) and that depressive symptoms (β = 0.881; 95% CI 0.541 to 1.220) and anxiety symptoms (β = 0.556; 95% CI 0.157 to 0.954) were lower the more frequently participants took part in angling. In terms of the normal length of an angling session, the only significant relationship was with the BDI score (β = −1.246; 95% CI −2.359 to −0.134), which conversely suggested that depressive symptoms were higher the longer the normal length of an angling session was.

## 4. Discussion

The main aim of the current study is to examine the relationship between recreational angling and mental health and well-being in a sample of UK adult male anglers. The results highlight that in terms of age-adjusted regression models, those who took part in angling more regularly were almost 17% less likely to report being diagnosed with depression, schizophrenia, suicidal thoughts, or having deliberately self-harmed compared to those taking part in angling less regularly. Also, those who took part in angling more regularly compared to those who did not were more likely to score highly on the WEMWBS (i.e., signifying higher mental well-being) and were less likely to score highly on the BDI and BAI (and therefore experience fewer depressive and anxiety symptoms). However, despite most results for the age-adjusted regression models being non-significant, suicidal thoughts and depressive symptoms were higher in those reporting a normal angling session lasting a longer period of time compared to a shorter period of time. Finally, this sample reported taking part in high levels of moderate and vigorous physical activity.

Given the potential health benefits of spending time in blue spaces [1,2], the findings in relation to improvements in specific aspects of mental health and mental well-being in those more frequently angling are not unexpected. A report exploring the health and well-being benefits of recreational angling in Western Australia provides some evidence to support our findings regarding the positive impacts on mental health and well-being [24]. This report highlights that one of the main motivations for angling in participants was relaxation and unwinding; 88.4% answered this was an important or very important reason. An important mediating variable to explain these motivations could be the time spent outdoors in blue spaces, with the median found to be 2 h per day in our current study. This could potentially be one of the reasons why the current study shows that, in general, mental health outcomes were better in recreational anglers who took part more frequently compared to those who took part less frequently. Notably, McManus and colleagues [24] highlighted that depression and anxiety were unlikely reasons to impact someone’s ability to participate in angling (0.6% and 0.4% reported these reasons, respectively). This provides a positive assurance that someone’s mental health status is unlikely to considerably impact their angling participation. However, it was also highlighted that some physical health problems such as back pain (3.9%), arthritis (2.8%), and knee/ankle problems (2.8%) were important barriers to angling participation. Therefore, future research in developing interventions and encouraging recreational angling in those with mental health disorders must also consider other comorbidities.

Putting the main findings into context, it is important to note that the prevalence of some aspects of mental health issues was higher when compared to the general UK population [25]. Examples include the prevalence of diagnosed anxiety disorder (16.1% vs. 5.9%), diagnosed depression (23.2% vs. 3.3%), and suicidal thoughts (30.3% vs. 20.6%) being more prevalent in the current study compared to the general UK population. However, the prevalence of bipolar disorder (0.9% vs. 2.0%), schizophrenia (0.4% vs. 0.5%), suicide attempts (6.8% vs. 6.3%), and deliberate self-harm (9.8% vs. 7.3%) were relatively similar between our study and the general UK population highlighted by McManus and colleagues [25]. The reasons for these differences are unclear, although they could be how the diagnosis of mental health issues was determined (i.e., the type of question asked) and also due to the specific characteristics of our study sample, which are not generalisable to the UK population. Some examples include being all male and mostly aged >45 years old, as well as having a lower employment rate (62.1%) compared with the UK population of 75.5% [26], although the levels of household income appear to be fairly representative [27]. Suicidal thoughts may be higher than the general UK population due to higher levels of diagnosed anxiety and depression, which were both linked to increased suicidal thoughts [28,29]. However, these comparisons are important because it demonstrates that despite relatively high numbers of individuals reporting diagnosed mental health, they have still chosen to participate in angling as their hobby. Indeed, some individuals may be using angling as a form of “self-therapy”, given the links between increased time spent in nature and psychological benefits [1,2,30]. In addition, a recent study highlighted that despite having some additional barriers to participation, those who considered themselves as having a disability were just as likely to take part in angling compared to those who did not [14]. They also highlighted that relaxing and enjoying the challenge of angling were key motivators for participation. This highlights that angling could be an equitable and inclusive activity for different groups to take part in. In particular, with males being less likely to take part in physical activity interventions [16] as well as having lower rates of social prescribing (64% females vs. 35% males) [31], this provides some evidence for angling’s potential role as a suitable option within social prescribing.

A survey conducted by Sport England in 2012 found that the drivers of satisfaction for general angling participants included the release and diversion as well as social aspects [32]. The key domains which emerged among these drivers included having the opportunity to ease stress; taking part in something different from the usual routine; the opportunity for a challenge; taking part in an activity without feelings of embarrassment/awkwardness; and not feeling threatened or intimidated. These identified drivers of satisfaction are not surprising considering a systematic review has highlighted that spending time in outdoor environments has positive impacts on both self-reported and physical biomarkers of stress [33]. Concurrently, a recent meta-analysis has highlighted that green exercise yields greater psychological advances compared to non-green environments [30]. In addition, this meta-analysis found that wild environments (where angling is likely to be practiced) may have further benefits in terms of improved vigour and comfort during green exercise [30]. These studies give further support for the development of angling-based interventions in individuals with mental health issues.

Another notable finding was that anglers reported taking part in 2.5 h/day of moderate–vigorous physical activity. Some of this specific activity is likely to be accumulated while angling, with estimated metabolic equivalent (MET) values for general angling activity being 3.5 METs, fishing from a riverbank being 3.5 METs, and fishing in a stream while in waders being 6.0 METs [34]. However, it must be recognised that self-reported physical activity time is likely to be overestimated compared with device-based measurement [35]. Therefore, it is necessary for future research to utilise more objective tools in order to more accurately determine the physical activity levels in male anglers. Also, further studies of a qualitative nature may be warranted to give extra context as to how male anglers may attribute taking part in angling as a way to improve their mental health and well-being.

The strengths of this study include recruiting a large sample size of male recreational anglers, using validated questionnaires to measure different facets of mental health such as anxiety and depressive symptoms. However, there are also limitations to consider. A cross-sectional study design was utilised, meaning causal relationships cannot be established between the different aspects of mental health and angling behaviour. Future research should utilise longitudinal designs to more accurately determine the association between mental health and recreational angling. Future research could also control for additional relevant covariates, such as education status and household income (measuring the latter variable using a continuous rather than categorical scale). This would help to further explain the associations between mental health and recreational angling. The normal length of an angling session was measured categorically, with participants only having four possible answer options. In combination, two of the options were chosen by only 0.8% of the sample. This limited the utility of this question, as an open answer option would have allowed for more of a spread of possible answers, and therefore, would have allowed to more definitively determine whether a longer time spent in a normal angling session promoted better mental health and well-being. This is likely to have resulted in some of the spurious results suggesting more time spent in a normal angling session was related to higher levels of suicidal thoughts and higher depressive symptoms. The views of those who were less digitally literate may have been missed due to using an online survey format [36]. The overall sample comprised males (although this is fairly representative of typical participation levels as recreational angling currently lacks female participation) [3,4]. However, this limits the inclusivity of the findings for the UK female population. The presence of psychiatric disorders was self-reported by participants; the linkage to a participant’s healthcare records would have more accurately determined this. Also, considering suicidal thoughts, suicidal attempts, and instances of self-harm were all self-reported, there is the potential for social desirability bias because some participants may be less likely to highlight suicidal thoughts and attempts due to the associated stigma [37].

In conclusion, this study has highlighted that those who took part in angling more regularly were less likely to report being diagnosed with depression, schizophrenia, suicidal thoughts, and having deliberately self-harmed compared to those taking part in angling less regularly. Taking part in angling more regularly also resulted in higher mental well-being and fewer depressive and anxiety symptoms compared to taking part less regularly. However, it should be noted that the anglers in this cohort appeared to have higher levels of diagnosed anxiety disorder, diagnosed depression, and suicidal thoughts than the general UK population. With this particular cohort self-reporting high levels of moderate–vigorous physical activity, this would suggest that encouraging participation in angling could be a good dual method strategy for both promoting relaxation and good mental health, as well as encouraging increased levels of physical activity within those with mental health issues, such as those experiencing suicidal thoughts. However, it will be necessary for future research to more clearly understand the exact mechanisms which explain the association between recreational angling and mental health. In addition to more traditional exercise prescriptions, such as walking and gym-based exercise, developing public health initiatives focused on promoting angling could provide a viable additional physical activity option for those with mental health issues.

## Figures and Tables

**Table 1:** Descriptive characteristics.

Variable	Percentage (n)/Median (25th–75th Interquartile Range)
Location (n = 1745)	
England	93.9% (1638)
Wales	2.8% (48)
Scotland	1.9% (33)
Northern Ireland	1.5% (26)
Ethnicity (n = 1750)	
White	99.1% (1735)
Black	0.2% (3)
Asian	0% (0)
Mixed/multiple	0.6% (10)
Other	0.1% (2)
Relationship status (n = 1748)	
Single	11.9% (208)
Married	62.9% (1110)
Widowed	1.4% (25)
Divorced	5.5% (97)
In a cohabiting relationship	17.7% (310)
In an open relationship	0.1% (1)
Other	0.4% (7)
Disability (n = 1745)	
Yes	16.0% (280)
No	84.0% (1465)
Employment status (n = 1749)	
Employed	50.6% (885)
Self-employed	10.7% (188)
Not in employment and looking for work	1.1% (20)
Not in employment and not currently looking for work	1.8% (31)
Homemaker	0.3% (5)
Student	0.7% (13)
Military	0.8% (14)
Retired	29.3% (512)
Unable to work	4.6% (81)
Household income (n = 1717)	
Below GBP 15,000	11.9% (205)
GBP 15,000 to GBP 25,000	21.1% (362)
GBP 25,000 to GBP 40,000	27.4% (471)
GBP 40,000 to GBP 60,000	19.1% (328)
Above GBP 60,000	20.4% (351)
Smoking status (n = 1749)	
Yes	18.8% (329)
No	81.2% (1420)
Alcohol status (n = 1725)	
Yes	66.0% (1139)
No	34.0% (586)
Psychiatric disorders	
Anxiety disorder (n = 1752)	
Yes	16.1% (282)
No	83.9% (1470)
Depression (n = 1752)	
Yes	23.2% (406)
No	76.8% (1346)
Bipolar disorder (n = 1752)	
Yes	0.9% (16)
No	99.1% (1736)
Schizophrenia (n = 1752)	
Yes	0.4% (7)
No	99.6% (1745)
Other psychiatric disorders (n = 1752)	
Yes	3.3% (58)
No	96.7% (1694)
Suicidal thoughts (n = 1752)	
Yes	30.3% (530)
No/Prefer not to say	69.7% (1222)
Suicide attempts (n = 1752)	
Yes	6.8% (118)
No/Prefer not to say	93.2% (1634)
Deliberate self-harm (n = 1752)	
Yes	9.8% (171)
No/Prefer not to say	90.2% (1581)
Mental health and well-being scores	
WEMWBS total score (n = 1715)	25 (21–28)
BDI total score (n = 1655)	7 (3–15)
BAI total score (n = 1552)	5 (1–13)
Angling status	
How often participants took part in angling (n = 1746)	
Every day	0.1% (1)
Five to six times per week	0.7% (13)
Three to four times per week	6.0% (104)
Once or twice a week	37.9% (662)
Once every two weeks	25.6% (447)
Once every month	16.4% (287)
Once every two or three months	8.9% (155)
Once every four, five, or six months	2.7% (47)
Less than once every six months	1.7% (30)
Normal length of an angling session (n = 1750)	
Under one hour	0.1% (1)
One to two hours	0.7% (20)
Three to four hours	13.1% (230)
Five or more hours	85.7% (1499)
Sedentary behaviour and physical activity	
Sitting time in hours/day (n = 1750)	2.0 (2.0–3.0)
Screen time in hours/day (n = 915)	2.0 (1.0–4.0)
Time spent outdoors hours/day (n = 1749)	2.0 (2.0–2.0)
MPA in hours/day (n = 912)	1.5 (1.0–4.0)
VPA in hours/day (n = 1724)	1.0 (1.0–2.0)

Data presented as percentages (number) for categorical variables and median (25th–75th interquartile range) for continuous variables unless otherwise stated; BAI = Beck Anxiety Inventory; BDI = Beck Depression Inventory; MPA = moderate physical activity; n = number; VPA = vigorous physical activity; WEMWBS = Warwick–Edinburgh Mental Well-being Scale.

**Table 2:** Logistic regression results showing relationship between angling status and psychiatric conditions after adjustment for age.

	How Often Participants Took Part in Angling		Normal Length of an Angling Session	
	Odds Ratio (95% CI)	*p*-Value	Odds Ratio (95% CI)	*p*-Value
Anxiety disorder	1.085 (0.986 to 1.195)	0.096	0.883 (0.643 to 1.213)	0.441
Depression	1.172 (1.078 to 1.273)	<0.001	0.800 (0.612 to 1.046)	0.102
Bipolar disorder	1.086 (0.757 to 1.559)	0.653	1.168 (0.295 to 4.628)	0.825
Schizophrenia	1.879 (1.177 to 2.997)	0.008	N/A (sample too small)	0.995
Other psychiatric conditions	0.993 (0.812 to 1.213)	0.942	1.185 (0.563 to 2.493)	0.655
Suicidal thoughts	1.162 (1.072 to 1.260)	<0.001	0.678 (0.521 to 0.882)	0.004
Suicide attempts	1.055 (0.916 to 1.214)	0.459	0.941 (0.586 to 1.512)	0.802
Deliberate self-harm	1.166 (1.034 to 1.314)	0.012	0.926 (0.608 to 1.410)	0.720

95% CI = 95% Confidence Interval.

**Table 3:** Linear regression results showing relationship between angling status and mental health and mental well-being after adjusting for age.

	How Often Participants Took Part in Angling		Normal Length of an Angling Session	
	β-Coefficient (95% CI)	*p*-Value	β-Coefficient (95% CI)	*p*-Value
WEMWBS score	−0.507 (−0.690 to −0.324)	<0.001	0.433 (−0.170 to 1.035)	0.159
BDI score	0.881 (0.541 to 1.220)	<0.001	−1.246 (−2.359 to −0.134)	0.028
BAI score	0.556 (0.157 to 0.954)	0.006	−0.887 (−2.171 to 0.398)	0.176

BAI = Beck Anxiety Inventory; BDI = Beck Depression Inventory; WEMWBS = Warwick–Edinburgh Mental Well-being Scale.

## Data Availability

The data that support the findings of the study are available from the corresponding author upon reasonable request.

## Supplementary Materials

The following supporting information can be downloaded at https://www.mdpi.com/article/10.3390/epidemiologia4030030/s1, Figure S1: fishing study online survey.
