# Supplementary material for: Mental Health and Recreational Angling in UK Adult Males: A Cross-Sectional Study
Source: Epidemiologia (Basel). 2023 Jul 13;4(3):298–308. doi: 10.3390/epidemiologia4030030 (PMC10366712; doi:10.3390/epidemiologia4030030)
Supplement: Supplementary file 1 [file epidemiologia-04-00030-s001.zip › epidemiologia-2358740-supplementary.pdf]

The Great British and Northern Ireland Fishing for Health Survey

Page 1: Page 1

The Great British and Northern Ireland Fishing for Health Survey

Overview

Recreational fishing is a sport and leisure activity in many countries worldwide and approximately 11% of the global population engage in recreational fishing. Fishing is plausibly linked to improved health through it being a therapeutic activity that is carried out in both blue and green space which have both been independently linked to better physical and mental health parameters.

There has been growing interest by recreational fishing groups and researchers in the health benefits of fishing. However, to date research in this area is limited, has been carried out in countries such as Australia, and no robust research exists that has been carried out in the UK. Moreover, little is known about why people engage in recreational fishing and why people do not engage.

The aim of this survey is to identify the physical and mental health benefits of recreational fishing in the UK and the barriers and facilitators to recreational fishing. This survey is a partnership between Anglia Ruskin University and Angling Direct. **You can take part if**

you are based in Great Britian or Northern Island and are over the age of 18.

What does taking part involve?

The research involves answering a set of survey questions and will take approximately 15 mins to complete.

Taking part in this survey is completely optional and you should only take part if you are willing to do so - you will be asked to confirm your consent to participate before you are able to begin the survey. There are no anticipated risk or precautions to be taken when participating in this study.

How will my information be used?

All information collected from you in relation to this research will be anonymised. Generated reports from this study will include your data but it will not be possible to identify you.

At the end of the survey, you will be asked if you are willing to be contacted by email for a follow-up interview. This is entirely voluntary and you do not have to provide us with any contact details or identifiable information.

By consenting to take part in this research you do not compromised your statutory legal rights. All information from your participation in this research will be collected and stored in accordance with the General Data Protection Regulation (GDPR 2016). By consenting to this study you are agreeing to Anglia Ruskin University processing personal data that you provide for any purpose connected to this study. Once you have consented, information from your submitted survey cannot be withdrawn

If you have any questions about taking part in the survey, please contact lee.smith@aru.ac.uk.

1. **Consent** In order to proceed, please tick the following six boxes to indicate your understanding and agreement. ✱ *Required*

Please select exactly 6 answer(s).

- [ ] I confirm that I have read the above information regarding the study, and understand what it involves.
- [ ] I understand how the information I provide in the study is to be used, and that it may be included in reports and publications.

- [ ] I understand that all information is anonymous, and I will not be identifiable.
- [ ] I understand that my participation in this study is completely voluntary, I am under no obligation to answer any questions, and I may stop the survey at any point without providing a reason.
- [ ] I understand that once I have completed the survey, I will not be able to retract my responses.
- [ ] I confirm that I am over the age of 18 and willing to take part in this study

Page 2: About You - Demographic Information

2. What is your age?

- 18 - 24 years old
- 25 - 34 years old
- 35 - 44 years old
- 45 - 54 years old
- 55 - 64 years old
- 65 - 74 years old
- 75 years and older

3. How would you describe your gender?

- Male
- Female
- Non-binary
- Intersex
- Other

3.a. If you selected Other, please specify:

4. Is your gender the same as your assigned sex at birth?

- Yes
- No

5. Where are you living?

- England
- Northern Ireland
- Scotland
- Wales

6. What is your ethnicity?

- White English/Welsh/Scottish/Northern Irish/British
- White Irish
- White Gypsy or Irish Traveller
- White non-British/ any other white background
- White and Black Carribean
- White and Black African
- White and Asian
- Any other mixed/multiple ethnic background
- Black British
- Black African
- Black Carribean
- Any other Black/African/Carribean background
- Arab
- Other

6.a. If you selected Other, please specify:

7. What is your relationship status?

- Single
- Married
- Widowed
- Divorced
- In a cohabiting relationship
- In an open relationship
- Other

7.a. If you selected Other, please specify:

8. Do you consider yourself to have a disability?

- Yes
- No

9. What is your current employent status?

- Employed
- Self-employed
- Not in employment and looking for work
- Not in employment and not currently looking for work
- Homemaker
- Student
- Military
- Retired

- Unable to work

10. What is your current **household** income?

- Below £15K
- £15K to 25K
- £25K to £40K
- £40K to £60K
- Above £60K

11. How many people live in your household?

- One
- Two
- More than two

Page 3: About You - Behaviours and Habits

12. Do you currently smoke?

- ○ Yes
- ○ No

12.a. If yes, how many cigarettes do you smoke on an average day?

[ ]

13. Do you drink alcohol?

- ○ Yes
- ○ No

13.a. If yes, how many units of alcohol do you consume on an average day?

[ ]

14. On the average day, how much time do you spend sitting?

[... hours and ... minutes]

15. On the average day, how much time do you spend outside?

... hours and ... minutes

Page 4: About You - Health and Wellbeing

Physical Activity

Think about all the vigorous and moderate activities that you do on an average day. Vigorous physical activities refer to activities that take hard physical effort and make you breathe much harder than normal. Moderate activities refer to activities that take moderate physical effort and make you breathe somewhat harder than normal.

16. On an average day, how much time do you spend doing moderate physical activity?

... hours and ... minutes

17. On an average day, how much time do you spend doing vigourous physical activity?

... hours and ... minutes

Screen Time

18. On an average day, how long do you spend looking at screens? (For example, computer, tablet or phone)

... hours and ... minutes

Physical Health

19. Have you ever been diagnosed with a physical health condition by a medical professional? Please select all that apply.

- [ ] No, I have never been diagnosed with a physical health condition.
- [ ] Obesity
- [ ] Hypertension
- [ ] Myocardial infarction
- [ ] Angina pectoris and other coronary diseases
- [ ] Other cardiac diseases
- [ ] Varicose veins of lower extremities
- [ ] Osteoarthritis
- [ ] Chronic neck pain
- [ ] Chronic low back pain
- [ ] Chronic allergy (excluding allergic asthma)
- [ ] Asthma (including allergic asthma)
- [ ] Chronic bronchitis
- [ ] Emphysema or chronic obstructive pulmonary disease (COPD)
- [ ] Diabetes type 1
- [ ] Diabetes type 2
- [ ] Diabetic retinopathy
- [ ] Cataracts
- [ ] Peptic ulcer disease
- [ ] Urinary incontinence or urine control problems
- [ ] Hypercholesterolemia
- [ ] Chronic skin disease
- [ ] Chronic constipation
- [ ] Liver cirrhosis and other hepatic disorders
- [ ] Stroke
- [ ] Chronic migraine and other frequent chronic headaches
- [ ] Hemorrhoids
- [ ] Cancer
- [ ] Osteoporosis
- [ ] Thyroid disease

- [ ] Renal disease
- [ ] Injury

Mental Health

20. Below are some statements about feelings and thoughts. Please tick the box that best describes your experience of each over the **last week.**

Please don't select more than 1 answer(s) per row.

	None of the time	Rarely	Some of the time	Often	All of the time
I’ve been feeling optimistic about the future	☐	☐	☐	☐	☐
I’ve been feeling relaxed	☐	☐	☐	☐	☐
I’ve been feeling useful	☐	☐	☐	☐	☐
I’ve been dealing with problems well	☐	☐	☐	☐	☐
I’ve been thinking clearly	☐	☐	☐	☐	☐
I’ve been feeling close to other people	☐	☐	☐	☐	☐
I’ve been able to make up my own mind about things	☐	☐	☐	☐	☐

21. Have you ever been diagnosed with a mental health condition by a medical professional? Please select all that apply.

- [ ] No, I have never been diagnosed with a mental health condition.
- [ ] Anxiety Disorder
- [ ] Depression
- [ ] Bipolar Disorder
- [ ] Schizophrenia
- [ ] Other psychiatric disorder

22. Have you ever thought of taking your own life, even if you would not actually really do it?

- ○ Yes
- ○ No
- ○ Prefer not to say

22.a. If yes, was this in the last month, last year, or at some other time?

- ○ In the last month
- ○ In the last year
- ○ At some other time

23. Have you ever made an attempt to take your own life?

- ○ Yes
- ○ No
- ○ Prefer not to say

23.a. If yes, was this in the last month, last year, or at some other time?

- In the last month
- In the last year
- At some other time

24. Have you ever deliberately harmed yourself in any way, but not with the intent of killing yourself?

- Yes
- No
- Prefer not to say

24.a. If yes, was this in the last month, last year, or at some other time?

- In the last month
- In the last year
- At some other time

Depression (Beck's Depression Inventory)

For each set of statements in this section, please select the one that is closest to how you feel.

25. Please select one answer:

- I do not feel sad
- I feel sad
- I am sad all the time and I can't snap out of it
- I am so sad and unhappy that I can't stand it

26. Please select one answer:

- I am not particularly discouraged about the future
- I feel discouraged about the future
- I feel I have nothing to look forward to
- I feel the future is hopeless and that things cannot improve

27. Please select one answer:

- I do not feel like a failure
- I feel I have failed more than the average person
- As I look back on my life, all I can see is a lot of failures
- I feel I am a complete failure as a person

28. Please select one answer:

- I get as much satisfaction out of things as I used to
- I don't enjoy things the way I used to
- I don't get real satisfaction out of anything anymore
- I am dissatisfied or bored with everything

29. Please select one answer:

- I don't feel particularly guilty
- I feel guilty a good part of the time
- I feel quite guilty most of the time
- I feel guilty all of the time

30. Please select one answer:

- I don't feel I am being punished
- I feel I may be punished
- I expect to be punished
- I feel I am being punished

31. Please select one answer:

- I don't feel disappointed in myself
- I am disappointed in myself
- I am disgusted with myself
- I hate myself

32. Please select one answer:

- I don't feel I am any worse than anybody else
- I am critical of myself for my weaknesses or mistakes
- I blame myself all the time for my faults
- I blame myself for everything bad that happens

33. Please select one answer:

- I don't have any thoughts of killing myself
- I have thoughts of killing myself, but I would not carry them out

- I would like to kill myself
- I would kill myself if I had the chance

34. Please select one answer:

- I don't cry any more than usual
- I cry more now than I used to
- I cry all the time now
- I used to be able to cry, but now I can't cry even though I want to

35. Please select one answer:

- I am no more irritated by things than I ever was
- I am slightly more irritated now than usual
- I am quite annoyed or irritated a good deal of the time
- I feel irritated all the time

36. Please select one answer:

- I have not lost interest in other people
- I am less interested in other people than I used to be
- I have lost most of my interest in other people
- I have lost all of my interest in other people

37. Please select one answer:

- I make decisions about as well as I ever could
- I put off making decisions more than I used to
- I have greater difficulty in making decisions more than I used to
- I can't make decisions at all anymore

38. Please select one answer:

- I don't feel that I look any worse than I used to
- I am worried that I am looking old or unattractive
- I feel there are permanent changes in my appearance that make me look unattractive
- I believe that I look ugly

39. Please select one answer:

- I can work about as well as before
- It takes an extra effort to get started at doing something
- I have to push myself very hard to do anything
- I can't do any work at all

40. Please select one answer:

- I can sleep as well as usual
- I don't sleep as well as I used to
- I wake up 1-2 hours earlier than usual and find it hard to get back to sleep
- I wake up several hours earlier than I used to and cannot get back to sleep

41. Please select one answer:

- I don't get more tired than usual
- I get tired more easily than I used to
- I get tired from doing almost anything
- I am too tired to do anything

42. Please select one answer:

- My appetite is no worse than usual
- My appetite is not as good as it used to be
- My appetite is much worse now
- I have no appetite at all anymore

43. Please select one answer:

- I haven't lost much weight, if any, lately
- I have lost more than five pounds
- I have lost more than ten pounds
- I have lost more than fifteen pounds

44. Please select one answer:

- I am no more worried about my health than usual
- I am worried about physical problems like aches, pains, upset stomach, or
- constipation
- I am very worried about physical problems and it's hard to think of much else

- I am so worried about my physical problems that I cannot think of anything else

45. Please select one answer:

- I have not noticed any recent change in my interest in sex
- I am less interested in sex than I used to be
- I have almost no interest in sex
- I have lost interest in sex completely

Anxiety (Beck's Anxiety Inventory)

46. Below is a list of common symptoms of anxiety. Please carefully read each item in the list. Indicate how much you have been bothered by that symptom during the past month, including today, by circling the number in the corresponding space in the column next to each symptom. For each question please indicate your answers as following: 0 = Not at all, 1 = Mildly, but it didn't bother me much, 2 = Moderately, it wasn't pleasant at times, 3 = Severely, it bothered me a lot

Please don't select more than 1 answer(s) per row.

	0	1	2	3
Numbness or tingling	☐	☐	☐	☐
Feeling hot	☐	☐	☐	☐
Wobbliness in legs	☐	☐	☐	☐
Unable to relax	☐	☐	☐	☐
Fear of worst happening	☐	☐	☐	☐
Dizzy or lightheaded	☐	☐	☐	☐
Heart pounding / racing	☐	☐	☐	☐
Unsteady	☐	☐	☐	☐
Terrified or afraid	☐	☐	☐	☐

Nervous	☐	☐	☐	☐
Feeling of choking	☐	☐	☐	☐
Hands trembling	☐	☐	☐	☐
Shaky / unsteady	☐	☐	☐	☐
Fear of losing control	☐	☐	☐	☐
Difficulty in breathing	☐	☐	☐	☐
Fear of dying	☐	☐	☐	☐
Scared	☐	☐	☐	☐
Indigestion	☐	☐	☐	☐
Faint / lightheaded	☐	☐	☐	☐
Face flushed	☐	☐	☐	☐
Hot / cold sweats	☐	☐	☐	☐

Social Network - Family

47. Considering the people you are related through birth, marriage, adoption and so on, how many relatives do you see or hear from at least once a month?

- ○ 0
- ○ 1
- ○ 2
- ○ 3 - 4
- ○ 5 - 8
- ○ 9 or more

48. Considering the people you are related through birth, marriage, adoption and so on, how many relatives do you feel at ease with that you can talk about private matters?

- 0
- 1
- 2
- 3 - 4
- 5 - 8
- 9 or more

49. Considering the people you are related through birth, marriage, adoption and so on, how many relatives do you feel close to such that you could call on them for help?

- 0
- 1
- 2
- 3 - 4
- 5 - 8
- 9 or more

Social Network - Friendship

50. How many of your friends do you see or hear from at least once a month?

- 0
- 1
- 2
- 3 - 4
- 5 - 8
- 9 or more

51. How many friends do you feel at ease with that you can talk about private matters?

- 0
- 1
- 2
- 3 - 4
- 5 - 8
- 9 or more

52. How many friends do you feel close to such that you could call on them for help?

- 0
- 1
- 2
- 3 - 4
- 5 - 8
- 9 or more

Loneliness

53. How often do you feel that you lack companionship?

- Hardly ever or never
- Some of the time
- Often

54. How often do you feel left out?

- Hardly ever or never
- Some of the time
- Often

55. How often do you feel isolated from others?

- Hardly ever or never
- Some of the time
- Often

Page 5: Questions About Fishing

56. Do you participate in fishing?

- Yes
- No

57. If no, please briefly state here why you do not currently participate in fishing.

If you selected no to the last question indicating that you do not currently participate in fishing, you have now reached the end of the survey questions. Please click through to the final page and select 'Finish' to submit your survey responses.

58. How often do you fish?

- Every day
- 5 or 6 times per week
- 3 - 4 times per week
- Once or twice at week
- Once every two weeks
- Once every month
- Once every two or three months
- Once every four, five or six months
- Less than once every 6 months

59. How long would you spend fishing on a normal fishing day?

- Under one hour
- 1 - 2 hours
- 3 - 4 hours
- 5 or more hours

60. Do you take part in fishing matches or competitions?

- Yes
- No

60.a. If yes, how often do you take part in fishing matches/competitions?

- Once or twice at week
- Once every two weeks
- Once every month
- Once every two or three months
- Once every four, five or six months
- Less than once every 6 months

61. What type of fishing do you do most often?

- Course fishing
- Sea fishing
- Match fishing
- Fly fishing
- Carp fishing
- Predator fishing

- Specialist fishing
- Other

61.a. If you selected Other, please specify:

62. What do you usually use to fish?

- Rod and line
- Pole/whip

63. How do you normally fish?

- Bait and wait (stationary)
- On the move (mobile)
- By boat

64. Where do you normally fish from?

- Lake bank
- River bank
- Beach/shoreline
- Boat
- Other

64.a. If you selected Other, please specify:

65. Approximately how long do you normally travel to go fishing?

- 10 minutes
- 20 minutes
- 30 minutes
- 40 minutes
- 50 minutes
- 1 hour,
- Between 1 hour and 1 hour 30 minutes
- Between 1 hour 30 minutes and 2 hours
- More than 2 hours

66. What is your main method of transport to get to a fishing venue?

- Car
- Bus
- Train
- On foot/ Walking
- By bike /Cycling
- Other

66.a. If you selected Other, please specify:

67. Are you more likely to fish alone or with someone else

- Alone
- With someone else

67.a. If you normally fish with someone else, are they your?

- Friend(s)
- Family member(s)
- Partner
- Not applicable

67.b. What is your main reason for going fishing?

- To socialise
- I enjoy the challenge of fishing
- To be outside
- To relax
- To catch food to eat
- Other

67.b.i. If you selected Other, please specify:

68. What are the main barriers that might stop you fishing? (You may select multiple)

- Bad weather

- [ ] Cost of fishing
- [ ] Lack of transport
- [ ] Lack of fishing venues nearby
- [ ] No one to go with
- [ ] Time restrictions
- [ ] Other

68.a. If you selected Other, please specify:

Page 6: Optional contact details - Follow up interview

69. If you would be willing to be contacted for a follow-up interview about fishing and wellbeing, please input your email address below.

Page 7: Final page

Thank you for taking part in this survey! If you have any questions or concerns, please contact lee.smith@aru.ac.uk

---
